# Probabilistic clinical studies and deterministic physiology: building a Monte Carlo bridge with the "Frank" project

**DOI:** 10.31744/einstein_journal/2026AE2239

**Published:** 2026-10-08

**Authors:** Felipe Coelho Argolo, Arthur Vasconcelos de Luna Freire, Larrie Rabelo Laporte, Guilherme Moreira Magnavita, Luís Cláudio Lemos Correia

**Affiliations:** 1 Universidade Federal da Bahia Hospital Universitário Professor Edgard Santos Departamento de Neurociências de Saúde mental Salvador BA Brazil Laboratório de Neuropsicofarmacologia (LANP), Departamento de Neurociências de Saúde mental, Faculdade de Medicina, Universidade Federal da Bahia, Hospital Universitário Professor Edgard Santos, Salvador, BA, Brazil.; 2 Universidade de São Paulo Instituto de Psiquiatria São Paulo SP Brazil Instituto de Psiquiatria, Universidade de São Paulo, São Paulo, SP, Brazil.; 3 Universidade Federal da Bahia Faculdade de Medicina da Bahia Salvador BA Brazil Faculdade de Medicina da Bahia, Universidade Federal da Bahia, Salvador, BA, Brazil.; 4 Universidade Federal de São Paulo Escola Paulista de Medicina São Paulo SP Brazil Escola Paulista de Medicina, Universidade Federal de São Paulo, São Paulo, SP, Brazil.; 5 Universidade de São Paulo Faculdade de Medicina Hospital das Clínicas São Paulo SP Brazil Instituto do Coração, Hospital das Clínicas, Faculdade de Medicina, Universidade de São Paulo, São Paulo, SP, Brazil.; 6 Emory University Rollins School of Public Health Department or Epidemiology Georgia United States Department or Epidemiology, Rollins School of Public Health, Emory University, Georgia, United States.; 7 Escola Bahiana de Medicina e Saúde Pública Salvador BA Brazil Escola Bahiana de Medicina e Saúde Pública, Salvador, BA, Brazil.

**Keywords:** Myocardial infarction, Probability, Physiology, Monte Carlo method, Cardiovascular agents, Cardiovascular diseases, Frank project

## Abstract

**Objective:**

To resolve the apparent paradox between deterministic physiological laws and probabilistic clinical outcomes by framing clinical variability as epistemic uncertainty within a complex, non-linear system, demonstrating how deterministic mechanisms can account for empirical distributions and outcome heterogeneity.

**Methods:**

We developed the "Frank" project, a 0-dimensional computational agent-based model (ABM) of human cardiovascular hemodynamics implemented in Julia (Agents.jl). Using Monte Carlo simulations (n=100), myocardial infarction was modeled by varying initial conditions, specifically sampling the infarction area from a Gaussian distribution along with systemic compliance. Cardiogenic shock was defined by a systolic arterial pressure <90mmHg and a cardiac index <2.2L/min/m^2^, with parameters evaluated via grid search against literature incidence benchmarks (5-10%).

**Results:**

The simulation produced hemodynamically stable, physiologically sound boundary parameters and pressure-volume loops. When initialized with a mean infarction area of 35±5% and systemic compliance of 1.5±0.3mL/mmHg, the model yielded a 7\% incidence of cardiogenic shock, closely matching empirical data. A strong linear inverse correlation was observed between infarction area and post-infarction systolic blood pressure (mean 93.96mmHg).

**Conclusion:**

Stochastic-appearing clinical trial outcomes can emerge from deterministic physiological systems characterized by sensitivity to initial parameters and hidden variables. The "Frank" framework establishes a Monte Carlo bridge between mechanistic physiology and epidemiological statistics, offering an approach to explore outcome heterogeneity and refine clinical trial interpretation and patient stratification.

## Highlights

From Galen to Otto Frank, haemodynamic models evolved in cardiac physiology.

In silico trials generate outcome distributions from varying physiological conditions.

Literature results on sudden death are reproducible with varied myocardial infarction areas.

## In Brief

Argolo et al. present a framework to solve the dichotomy between probabilistic concepts in clinical studies and the underlying deterministic physiology. By conducting an in-silico trial, they simulate conditions for sudden cardiac death, obtaining statistical results through variation of physiological configurations (infarction sizes).

## Graphical abstract

**Figure:**
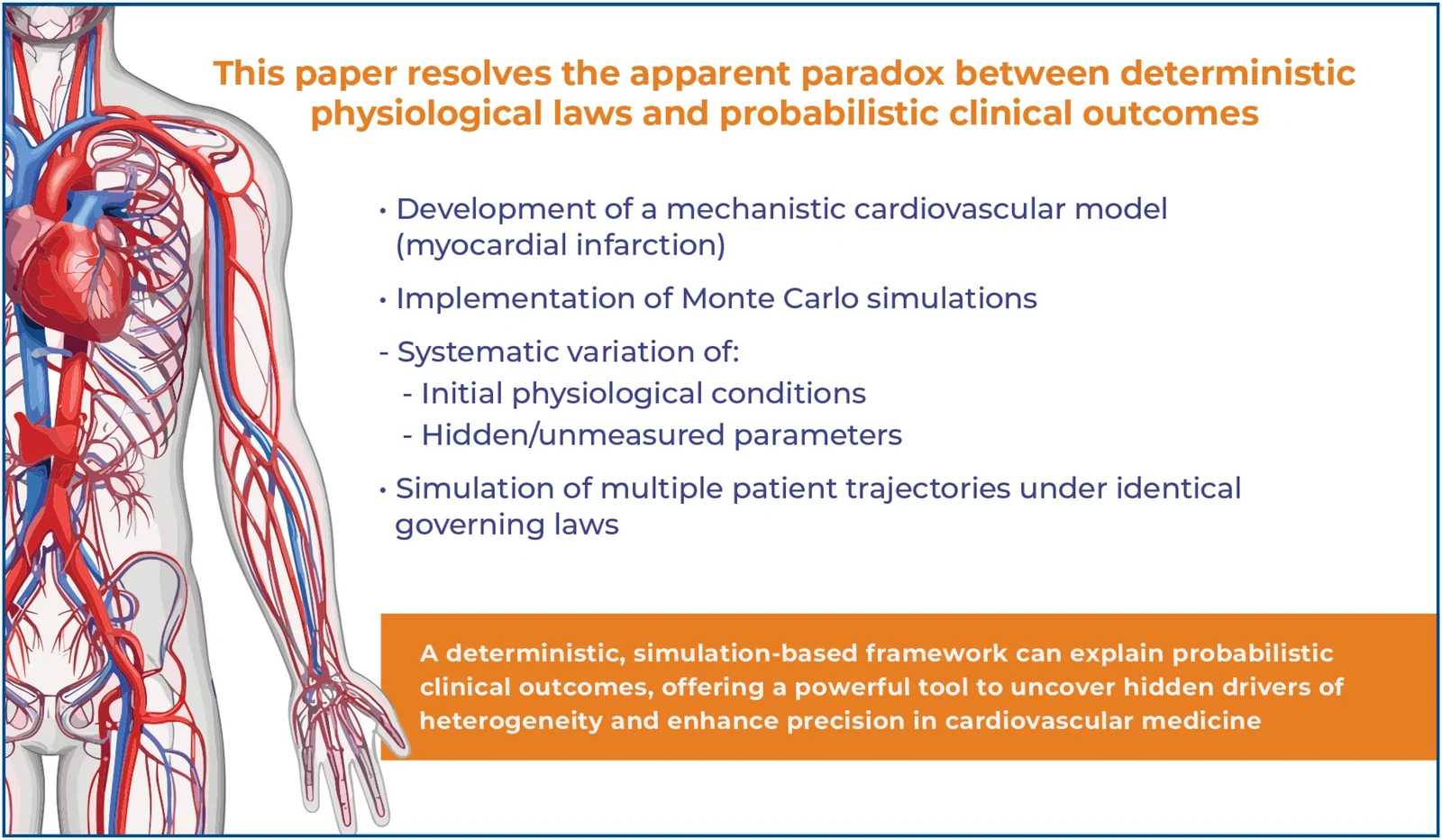

## INTRODUCTION

Since its inception, the concept of disease has considered heterogeneous presentations of clinical symptoms. Early descriptions already recognize the slight variations among patients with the same diagnosis.^(1)^ These reflect individual variations, such as genetics or environmental factors, and also unknown factors that influence human health.^(1,2)^ This contrasts with the deterministic nature of the underlying processes, suggesting that there is a gap between mechanistic physiological descriptions and their final manifestation as diseases.^(3)^

The advent of clinical trials and recent mathematical models made it possible to summarize information from heterogeneous measures using abstractions from statistics and probability theory.^(4)^ This was extremely useful, as it allowed progress in the measurement of effects without a complete picture of the underlying biology. However, this method may contain a caveat: the hidden parts of the disease phenomenon are understood through pure probabilistic models.^(3)^ This leaves variability untouched by clinical studies, resulting in a gap between physiopathological models and clinical presentation.

This paper examines this philosophical tension and proposes a methodology to reconcile deterministic physiology with probabilistic clinical outcomes. These devices bring to light (a) the physiological models underlying clinical questions and (b) the plausibility of different explanations contributing to outcome heterogeneity.

### Historical context

The understanding of cardiovascular physiology has evolved over centuries, moving progressively from philosophical speculation to precise, quantitative models. Early theories, such as those by Galen of Pergamon, were speculative, proposing concepts like invisible pores in the interventricular septum and describing the heart as a "furnace".^(5)^ A significant shift toward a mechanistic and observational approach occurred with Ibn al-Nafis, who correctly described pulmonary circulation, refuting Galen by detailing the blood's passage through the lungs;^(6)^ This transition was solidified by William Harvey, whose experimental work in *De Motu Cordis* established the systemic circulation as a closed system, with the heart acting as a central pump, laying the foundation for modern cardiovascular physiology.

Following the establishment of this experimental groundwork, the focus shifted towards quantifying physiological processes. This era produced foundational quantitative models that are still used today. The idea of haemodynamic compartments as compressible vessels of air (the Windkessel model) was alluded to by Giovanni Borelli, and Stephen Hales articulated the concept more clearly, drawing the analogy with fire engine air chambers.^(6)^ Otto Frank provided a mathematical framework for the Windkessel, precisely describing arterial compliance and pressure damping.^(7)^ The Frank-Starling law established a clear relationship between diastolic filling and the force of contraction.^(8,9)^ The Fick principle offered a method to calculate cardiac output based on oxygen consumption.^(10)^ These developments, along with Bernard's concept of homeostasis,^(11)^ marked a pivotal move towards viewing the cardiovascular system as a predictable, deterministic machine governed by physical laws.

More recently, particularly over the last century, clinical decision-making has been dominated by a probabilistic perspective supported by epidemiology. The popularization of clinical studies to quantify outcomes led to widespread use of numerical estimates, introducing concepts from statistics and probability into medical terminology. While this approach has been invaluable for managing uncertainty and assessing interventions at a population level, it has also shifted the focus away from investigating the underlying deterministic mechanisms. Consequently, a gap has emerged: the probabilistic findings of clinical studies often remain disconnected from the deterministic principles of physiology, leaving the heterogeneity observed in patient outcomes largely unexplained.

### The deterministic-probabilistic paradox in clinical sciences

Clinical medicine faces a fundamental epistemological challenge: while biological systems operate according to deterministic physical and chemical laws, clinical outcomes are typically reported as probabilities. This creates an interpretive dilemma: do these probabilities represent genuine randomness at the individual level, or merely our incomplete knowledge of the deterministic processes at work?

Considering the underlying mechanistic properties of physiological processes, we assume that, unlike sub-atomic phenomena, disease processes are not fundamentally indeterminate, with a few possible exceptions.^(12)^ That is, variations in clinical outcomes can be better understood as manifestations of deterministic chaotic systems and the presence of hidden variables, in which sensitivity to initial conditions and unknown factors create the appearance of randomness within fundamentally deterministic processes.^(13, 14)^

Chaotic systems exhibit extreme sensitivity to initial conditions, where infinitesimal differences in starting parameters can lead to dramatically divergent outcomes.^(15)^ In cardiovascular physiology, for instance, millisecond differences in ventricular contraction timing relative to other cardiac events can cascade into radically different hemodynamic outcomes.^(16)^ In addition, unmeasured or unaccounted mechanisms may significantly influence the observed outcomes. Altogether, chaotic dynamics and hidden factors lead to a heterogeneous set of observations, which is often modelled through probability and statistics. Our work suggests a framework that bridges probabilistic clinical models and deterministic physiological ones.

From an ontological perspective, we assume that macroscopic physiological processes are largely deterministic. Unlike quantum phenomena, which may exhibit ontological indeterminism, cardiovascular dynamics are governed by classical physics. Thus, the variability observed in clinical outcomes is best understood as epistemic probability, a measure of our uncertainty regarding the precise initial conditions and hidden variables of the individual patient.^(17)^

### Frank: Taking the Monte Carlo road from deterministic physiology to statistics of clinical outcomes

When planning a clinical trial, one should be able to model a plausible disease pathophysiology that may lead to the expected outcomes. On the other hand, one should also be able to explain the variance in responses according to candidate hidden variables. In essence, our framework uses Monte Carlo methods^(18)^ to bridge the probabilistic quantities observed in clinical studies and the physiological models that guide medical practice.

A brief history of Monte Carlo methods goes back to William Thomson ("Lord Kelvin"),^(19)^ who used cards and coins to sample from distributions related to the kinetics of gases. Later, Stanislaw Ulam, while playing solitaire, envisioned that it was plausible to lay out a deck many times and count the successful plays to empirically study the number of good games. John von Neumann helped implement Ulam's statistical approach on a computer, enabling it to calculate the evolution of hundreds of deterministic systems. Shortly after, Nick Metropolis, a fellow Manhattan Project physicist, coined the term "Monte Carlo Method".^(20)^

Current medical practice makes use of several abstractions with probabilities in their definition. Ideas such as ‘relative risks’ and ‘odds ratios’ are frequently used by health practitioners, while clinical studies are deployed to produce numeric estimates of these quantities. Nonetheless, the connection between epidemiological measures and the quantities used to describe diseases is thin. A doctor will often reason using information from physical examination and physiopathological models. Epidemiological information is only considered afterwards, to estimate the magnitude and heterogeneity of effects.

This study illustrates the use of Monte Carlo models to reframe statistical distributions and probabilities as long-term outcomes in physiological systems with slightly different characteristics. In essence, our framework uses Monte Carlo methods not to introduce ontological randomness into the physiology but to propagate the epistemic uncertainty of the population-level down to the deterministic physiological level.

## OBJECTIVE

Frank (a homage to Otto Frank) is a proof-of-concept project that investigates Myocardial Infarction. In particular, we try to understand whether our physiological models of disease can explain the distributions empirically observed in clinical trials. In addition, whether we can identify which hidden factors or initial conditions better explain outcome heterogeneity once trial results are reported.

## METHODS

### Rationale and study design

The core of this study is a computational agent-based model (ABM) of the human cardiovascular system. Unlike statistical models that infer correlations from aggregate data, this generative model aims to demonstrate the sufficiency of specific physiological mechanisms to produce the emergent phenomenon of cardiogenic shock. It serves as an *in silico* laboratory to test mechanistic hypotheses.

In this simulation, at each time step, haemodynamic components interact. For instance, blood can flow from cardiac chambers to the pulmonary and cardiac circulation. The study includes an in-silico trial^(21,22)^ that initializes a Myocardial Infarction clinical case with slightly different conditions (e.g. infarction area). A hundred patients were analyzed with Monte Carlo methods to determine clinical outcomes based on physiological thresholds. The simulation was implemented in the Julia programming language (v1.6) with its core built upon the Agents.jl package.

### The "Frank" cardiovascular simulation model

The "Frank" model is a minimalistic 0-dimensional ABM of human hemodynamics, simulating cascading events throughout the system, composed of connected compartments (Figure 1). The pacemaker starts each heart contraction, leading the pressure gradients at each consecutive compartment to drive blood flow in a cyclic manner. The components (agents listed below) have intrinsic properties, such as baseline volume and elastance. A list of agents includes: Heart Chambers: Agents that pump blood to the systemic and pulmonary circulation; Pulmonary circulation: Circulating blood coming out of the Right Ventricle; Vascular Compartments: Blood vessels based on the Windkessel model, including resistance, pressure and elastance; Pacemaker: A Sinoatrial node agent initiating the cardiac cycles; Coronary circulation: A module linking systemic pressure to myocardial perfusion.

**Figure 1:**
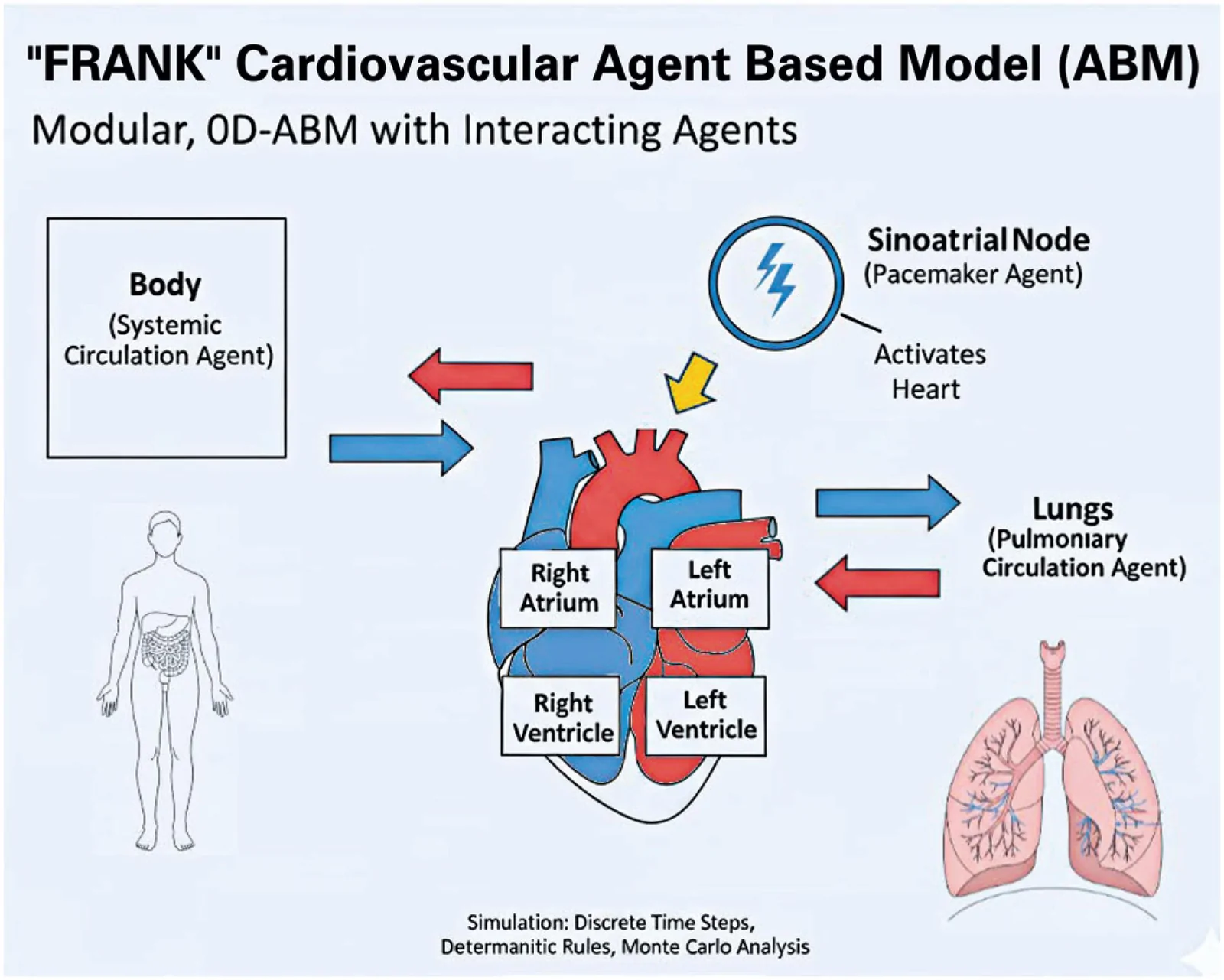
Diagram of an agent-based model including main components. The sinoatrial node initiates each cycle, prompting the atria and ventricles to contract (systole), circulating blood towards the lungs and the body. The diastolic phase allows the heart chambers to be filled with blood

Source: The illustrations were sketched using generative AI (Google Gemini). They were made by me with the open-source software GIMP: manual edits to color, anatomical corrections, repositioning of image elements, diagrams (arrows, boxes, etc.) and fonts/text created manually.

Simulation progresses in discrete time steps, where the state of all agents is updated based on deterministic physiological rules. Pathological conditions, such as Myocardial Infarction, are introduced by altering agent properties. To generate statistical distributions, we conduct Monte Carlo simulations by sampling key initial parameters from predefined physiological distributions. The outcome of cardiogenic shock was defined when the simulation reached a state of critical circulatory collapse, identified by a systolic arterial pressure falling below 90mmHg and a cardiac index <2.2L/min/m^2^ (cardiac output/body surface). These parameters indicate severe systemic hypoperfusion, associated with 1-year mortality rates close to 50%.^(23)^

Detailed specifications of the model are presented in table 1. We ran simulations with 100 patients and identical conditions, except for the size of infarction area, which followed a random Gaussian distribution. Empirical results were probed with grid search to find appropriate parameters (mean μ, std. dev. σ) that led to conditions consistent with literature-reported cardiogenic shock incidence (5-10%).^(23)^

**Table 1:** Cardiovascular simulation model

Variable	Function	Parameters
Ventricular pressure	$P_{v}(t)=e(t)\cdot \left(V_{v}(t)-V_{d}\right)$	e(t): Elastance at time t, V_0_: unstressed volume, V_v_(t): ventricular volume at time t
Elastance	$e(t)=E_{min}+\left(E_{max}-E_{min}\right)\cdot (t)$	E_max_: Maximum elastance, Emin: Minimum elastance, α): Periodic activation function
Systemic flow	$Q_{\text{sys}}=P_{\text{p}}-P_{\text{d}}/R_{\text{sys}}$	P_p_: Proximal pressure, P_d_: Distal pressure, R_sys_: Systemic vascular resistance
Volume change	$\frac{{\text{dV}}_{\text{v}}}{\text{dt}}=Q_{\text{in}}-Q_{\text{out}}$	V_v_: Ventricular Volume, Q_in_: Volume entering the Ventricle, Q_out_: Volume leaving the Ventricle
Vascular compliance	$\text{C}=\frac{\text{P}}{\text{V}-{\text{V}}_{\text{u}}}$	C: Compliance, P: Pressure, V: Total volume, V_u_: Unstressed volume
Contractility loss	$E_{post}=E_{pre}\cdot \left(1-I_{s}\right)$	E_mp_: Maximum Elastance post myocardial infarction, E_mpr_: Maximum Elastance pre myocardial infarction, I_s_: Percentage of the myocardium affected by infarction

Source: Authors. The table refers to data generated in the simulations performed in this study.

## RESULTS

### Simulation stability and boundary conditions

The simulation presented parameters within physiological ranges and displayed qualitatively adequate curves (e.g. closed LV pressure-volume loops). Descriptive plots and summarized data for a single patient trial are displayed in figure 2 and table 2. The implemented simulations showed results that complied with physiological boundaries.

**Figure 2:**
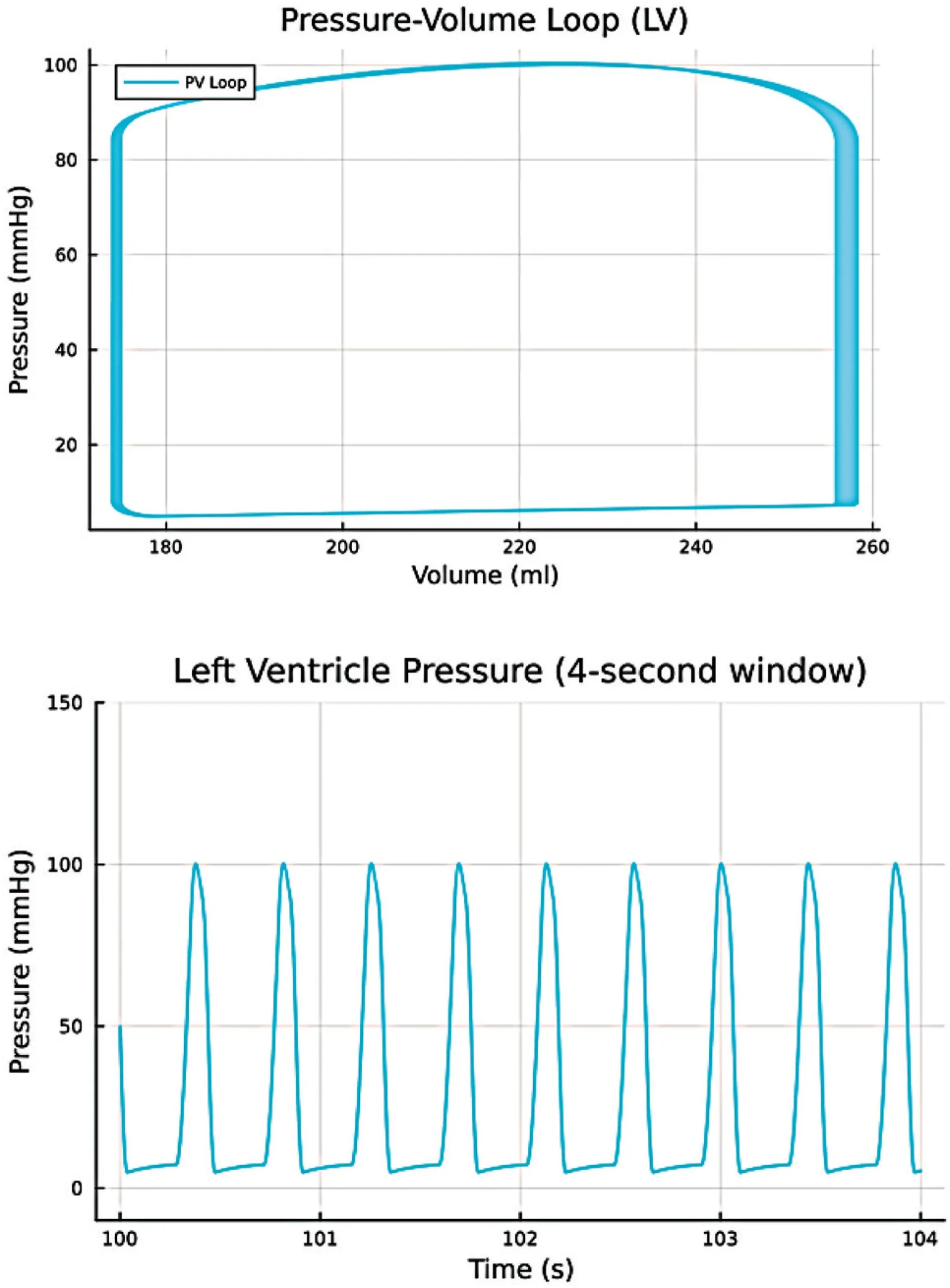
Pressure/Volume loop of the left ventricle (left) & pressure on left ventricle measured over 4 cardiac cycles (right)

**Table 2:** Descriptive statistics of haemodynamics parameters under regular and MI conditions

Measure 1st row - Regular condition 2nd row - MI condition	Min / Max	Mean±SD	Median (Q1, Q3)
Systolic blood pressure	116.69 / 117.71 79.18 / 84.13	117.22±0.11 82.62±1.58	117.23 (117.18, 117.26) 83.23 (81.26, 84.08)
Diastolic blood pressure	74.21 / 74.21 59.11 / 67.63	74.21±0.00 61.91±2.71	74.21 (74.21, 74.21) 61.02 (59.31, 64.26)
Mean arterial pressure	88.37 / 88.71 65.95 / 73.10	88.55±0.04 68.81±2.26	88.55 (88.54, 88.56) 68.43 (66.61, 70.86)
Systolic pulmonary arterial pressure	20.68 / 20.75 68.70 / 69.86	20.69±0.01 69.27±0.31	20.69 (20.69, 20.69) 69.24 (68.97, 69.54)
Diastolic pulmonary arterial pressure	14.54 / 14.54 25.00 / 25.00	14.54±0.00 25.00±0.00	14.54 (14.54, 14.54) 25.00 (25.00, 25.00)
Mean pulmonary arterial pressure	18.68 / 19.06 39.57 / 39.95	18.92±0.12 39.76±0.10	18.95 (18.82, 19.03) 39.75 (39.66, 39.85)
Pulmonary resistance	0.10 / 0.10 0.25 / 0.25	0.10±0.00 0.25±0.00	0.10 (0.10, 0.10) 0.25 (0.25, 0.25)
Systematic resistance	1.00 / 1.00 1.65 / 1.75	1.00±0.00 1.70±0.04	1.00 (1.00, 1.00) 1.70 (1.66, 1.74)
Central venous pressure	5.00 / 5.00 15.80 / 15.85	5.00±0.00 15.83±0.01	5.00 (5.00, 5.00) 15.83 (15.82, 15.84)
Heart rate	60.00 / 60.00 142.45 / 154.67	60.00±0.00 148.59±3.53	60.00 (60.00, 60.00) 148.60 (145.53, 151.64)

The MI condition resulted in increased pulmonary pressure (systolic, diastolic and mean pressure), pulmonary/systemic resistance, central venous pressure and heart rate. Conversely, the systemic arterial blood pressure (systolic, diastolic and mean pressure) was reduced. Considering a 50% reduction of coronary perfusion to the heart, MAP fell below the threshold chosen to define cardiogenic shock outcome (<90mmHg; Table 2, entries in 2nd row).

### Clinical validation

Depending on the initial parameters, myocardial infarction presented different clinically measurable outcomes. When running the simulation for multiple samples with different initial conditions, we were able to reproduce the observed percentages of cardiogenic shock. Our model was able to replicate this outcome under a specific range of initial conditions representing this high-risk population.

The grid search results suggested that acceptable conditions were achieved with a mean infarction area equal to 35% of the total (μ=35) and standard deviation equal to 5% (σ=5). The second parameter, systemic compliance, was set to a mean of 1.5ml/mmHg and standard deviation of 0.3ml/mmHg. These parameter values yielded a 7% cardiogenic shock incidence, within the predefined interval (5-10%). In the simulated haemodynamics, this means that blood flow was compromised by 35%, leading to a dampened contraction that corresponded to a fraction of an unaffected heart pump. Post-infarction average systolic blood pressure was 93.96mmHg and there was an almost perfectly linear correlation with infarction area (Figure 3).

**Figure 3:**
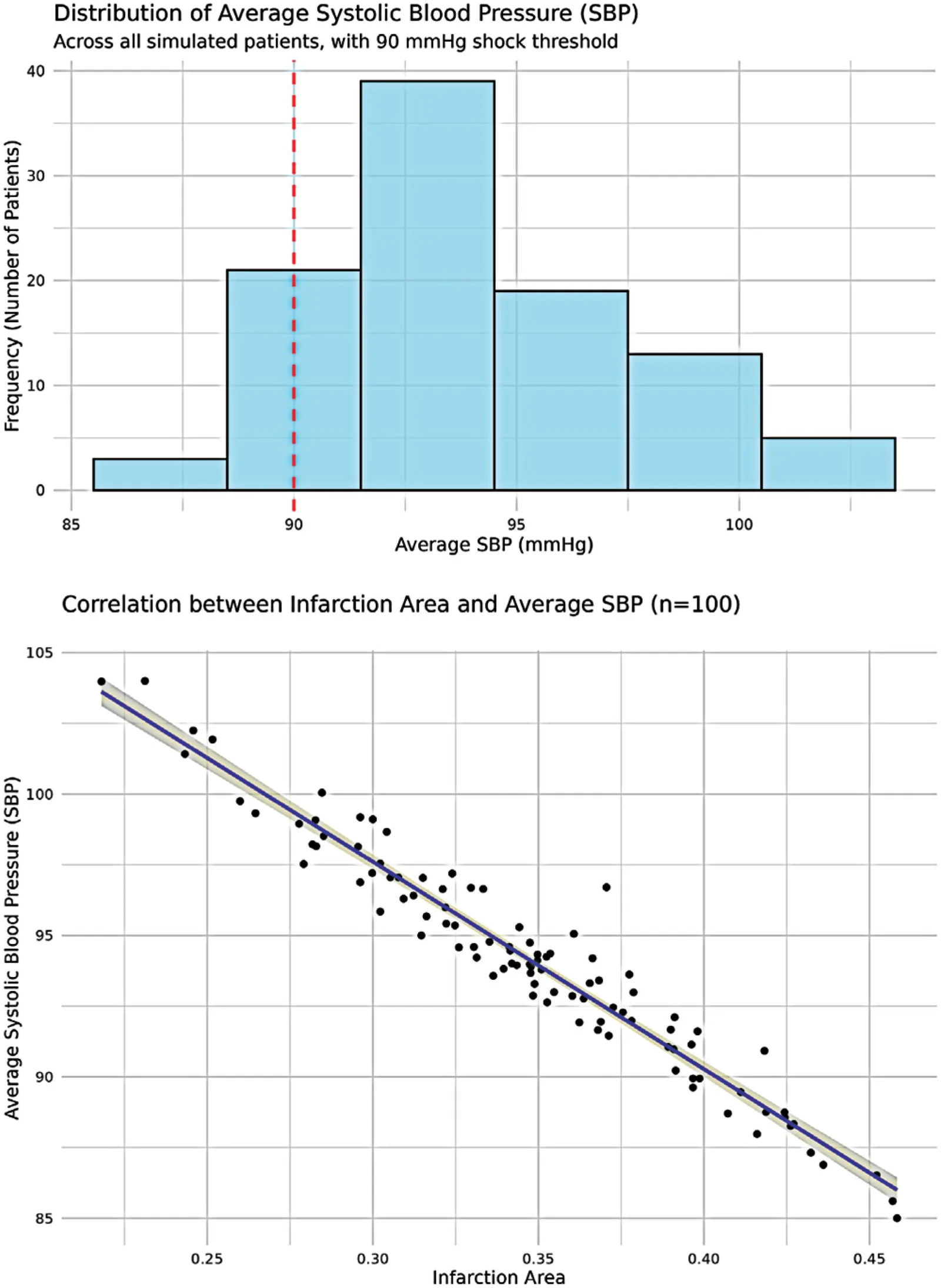
Top row shows a histogram of average systolic blood pressure (SBP), including the 90mmHg threshold for cardiogenic shock. The bottom row displays a scatter plot between infarction area and average SBP

## DISCUSSION

Our approach directly addresses the call for evidence pluralism in medicine, notably encapsulated in the Russo-Williamson thesis: that probabilistic evidence needs to be accounted for by an underlying mechanism before the causal claim can be established.^(24)^ By integrating mechanistic evidence (the deterministic ABM) with probabilistic evidence (clinical trial data), we provide a more robust causal assessment than either method could achieve in isolation. The simulation demonstrates that the statistical associations observed in epidemiology are grounded in the productive continuity of physiological mechanisms. To establish causal claims, scientists need the mutual support of mechanisms and dependencies.

In medicine, physiology follows a deterministic logic. However, the corresponding pathological models assume heterogeneity owing to intrinsic randomness or loosely defined conditions. The conceptual gap between deterministic physiological models and probabilistic clinical outcomes represents a significant barrier to advancing mechanistic understanding of disease.

The introduction of generative models to account for chaotic sensitivity and putative hidden variables establishes a new paradigm for clinical investigation. This approach brings the mathematical rigor of physics to medical research, potentially transforming how we understand, classify, and treat complex conditions. By explicitly modeling how small variations in initial conditions or unmeasured variables can generate divergent outcomes, we provide a mechanistic framework for explaining clinical heterogeneity.

Our simulation approach can identify physiological parameters for patient stratification in future trials. By identifying parameters with high sensitivity, researchers can prioritize measurements that reduce unexplained variability, potentially improving statistical power and leading to more personalized treatment recommendations.^(25)^

While the parameter set (Infarction Area *μ*=35%, σ=5%) successfully reproduces the empirical incidence of cardiogenic shock reported by Samsky and colleagues,^(23)^ we acknowledge the potential for model underdetermination. Other combinations of physiological models and different parameter choices might yield similar aggregate outcomes. As different sampling procedures can yield identical distributions, distinct generative models can describe the same complex phenomena from different perspectives.

The main role of our models is to establish and test the assumptions behind a disease model. The point is not to clarify the exact nature of putative mechanisms, but to verify the plausibility of such assumptions and the conditions under which they hold true. The method helps to pinpoint the mechanisms, workarounds, and parameter variations needed to explain observations from clinical studies.

## CONCLUSION

This paper presents a novel methodological framework for understanding heterogeneity in clinical trial outcomes through the lens of deterministic chaos in cardiovascular physiology. By demonstrating how probabilistic outcomes can emerge from deterministic systems that are sensitive to initial conditions, we provide a mechanistic approach to explaining and potentially predicting variable treatment responses. The proof-of-concept application to myocardial infarction demonstrates that seemingly random variations can be explained through specific physiological parameters. By bridging the philosophical divide between deterministic physiology and probabilistic clinical outcomes, this methodology offers a promising path toward more mechanistically grounded clinical research.

## Data Availability

The content is already available.
